# Supplementary material for: GeneFEAST: the pivotal, gene-centric step in functional enrichment analysis interpretation
Source: Bioinformatics. 2025 Mar 3;41(3):btaf100. doi: 10.1093/bioinformatics/btaf100 (PMC11919446; doi:10.1093/bioinformatics/btaf100)
Supplement: btaf100_Supplementary_Data [file btaf100_supplementary_data.pdf]

GeneFEAST: the pivotal, gene-centric step in functional enrichment analysis interpretation

Supplementary Information

Avigail Taylor[1,2,3 *], Valentine M Macaulay[3†], Matthieu J Miossec[2], Anand K Maurya[1] and Francesca M Buffa[4]

[1]Nuffield Department of Medicine, University of Oxford, Old Road Campus, Oxford, OX3 7BN, [2]Centre for Human Genetics, University of Oxford, Roosevelt Drive, Oxford, OX3 7BN, [3]Nuffield Department of Surgical Sciences, University of Oxford, ORCRB, Roosevelt Drive, Oxford, OX3 7DQ, [4]Computational Biology & Integrative Genomics Lab, Department of Oncology, University of Oxford, ORCRB, Oxford, OX3 7DQ

[†]Deceased.

*To whom correspondence should be addressed.

Section 1. The landscape of existing functional enrichment analysis tools

Web-based versus standalone functional enrichment analysis tools

Supplementary Figure 1 depicts the overall functional enrichment analysis (FEA) workflow, and the FEA tools currently available. (See Supplementary Table 1 for the citation or website for each of the tools depicted). In addition to categorising tools according to their functionality, tools are also grouped by their implementation, i.e. into web-based (WB) and standalone (SA) tools. SA tools can be further categorised according to their interface, which can be command-line (CL), or graphical user interface (GUI) (see Supplementary Table 2).

How a tool is implemented impacts the way in which it can be used: WB tools are widely considered to be easier to use than locally installed, SA tools, with few learning barriers in the way of the non-computational user. In addition, WB tools can be run from powerful servers, where storage space for large databases is not a concern, and neither is computational power required for analyses. In contrast, SA tools, especially those with a CL interface (SA-CL), often require some level of computational ability on the part of the user, which can represent a barrier to use. However, SA tools have several advantages over WB tools: (1) SA tools are not a shared resource, so do not suffer from the associated bottlenecks to computation; (2) SA-CL tools can be used in a flexible, modular way and can be incorporated into bespoke, automated computational pipelines, making them ideal for use with multiple, large datasets. Note, however, that web services can also be used in this manner when they provide an application programming interface in addition to the primary website.; (3) When SA tools are published under an Open Source licence, the expert user can modify such tools as required for their needs.

GeneFEAST is an SA-CL summarisation and visualisation tool for summarising and visualising FEA results arising from any standard ’omics database of terms and upstream FEA pipeline.

Functionality provided by existing FEA summarisation tools

Supplementary Table 2 lists FEA tools that provide a summarisation step, and the additional features that they provide beyond basic summarisation of FEA results.

Supplementary Figure 1

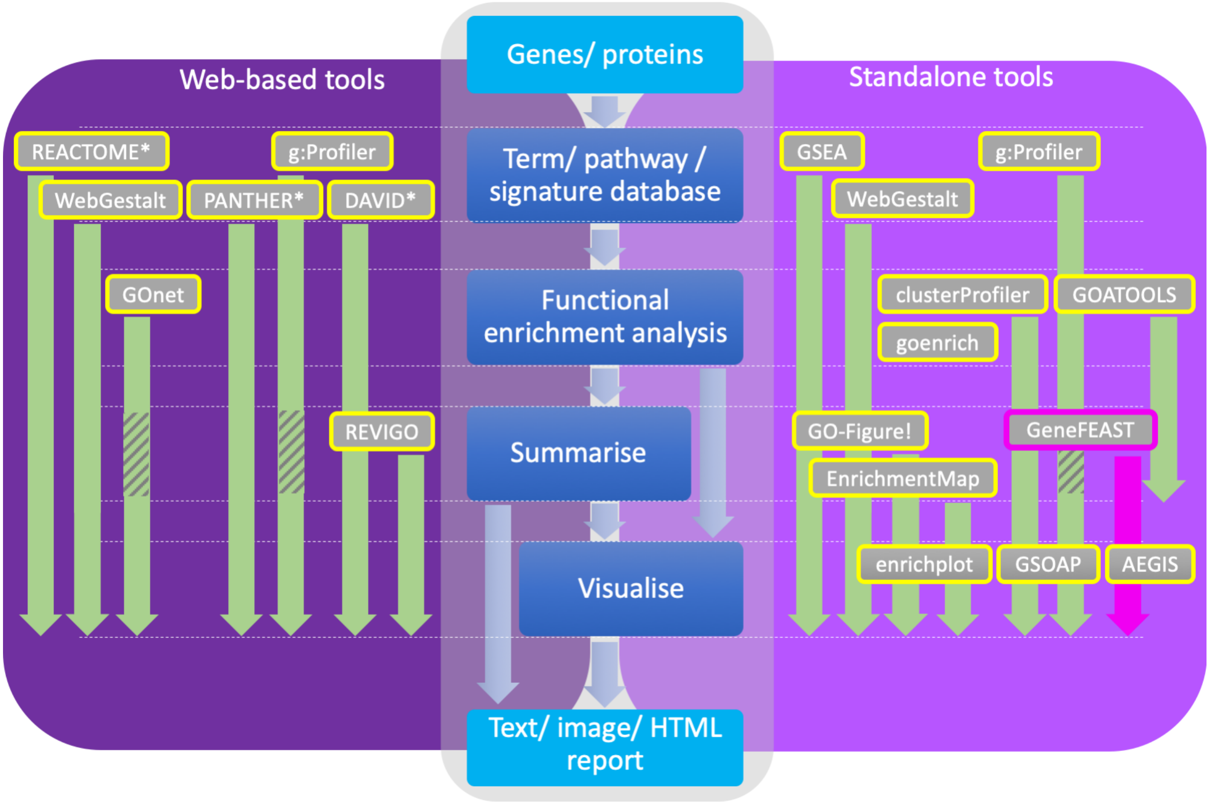

Supplementary Figure 1. The functional enrichment analysis (FEA) workflow, and the landscape of tools available. Tools shown here are representative of those available, but do not comprise an exhaustive list. Horizontally, tools are categorised by the functions that they perform. In addition, tools are split into web-based (left, dark purple) and standalone tools (right, light purple); tools appearing on both sides of the figure are available in both a web-based and standalone format. Some web-based tools provide a programmatic interface via an application programming interface, these are denoted with a '*'. Where an arrow is hashed out in grey, the corresponding tool does not perform that part of the FEA workflow. GeneFEAST is highlighted in pink. Please note: by definition, all tools that perform the 'Functional enrichment analysis' step link up to some database of terms, pathways or signatures; however, only those tools that provide either a novel database, or a significant curation of existing databases, are shown in the 'Term/ pathway/ signature database' step. Citations or websites for tools shown here are given in Supplementary Table 1.

Supplementary Table 1

Tool	Citation or website
**AEGIS**	Zhu, J.*, et al.* Exploratory Gene Ontology Analysis with Interactive Visualization. *Sci Rep* 2019;9(1):7793.
**clusterProfiler**	Wu, T.*, et al.* clusterProfiler 4.0: A universal enrichment tool for interpreting omics data. *The Innovation (N Y)* 2021;2(3):100141.
**DAVID**	Huang, D.W.*, et al.* The DAVID Gene Functional Classification Tool: a novel biological module-centric algorithm to functionally analyze large gene lists. *Genome Biol* 2007;8(9):R183.
**EnrichmentMap**	Enrichment map: a network-based method for gene-set enrichment visualization and interpretation. *PLoS One* 2010;5(11):e13984.
**enrichplot**	Yu G. *enrichplot: Visualization of Functional Enrichment Result*. R package version 1.14.1, https://yulab-smu.top/biomedical-knowledge-mining-book/.
**GOATOOLS**	Klopfenstein, D.V.*, et al.* GOATOOLS: A Python library for Gene Ontology analyses. *Sci Rep* 2018;8(1):10872.
**goenrich**	https://github.com/jdrudolph/goenrich
**GO-Figure!**	Reijnders, M.J.M.F. and Waterhouse, R.M. Summary Visualizations of Gene Ontology Terms With GO-Figure! *Frontiers in Bioinformatics* 2021;1(6).
**GOnet**	Pomaznoy, M., Ha, B. and Peters, B. GOnet: a tool for interactive Gene Ontology analysis. *BMC Bioinformatics* 2018;19(1):470.
**g:Proflier**	Raudvere, U.*, et al.* g:Profiler: a web server for functional enrichment analysis and conversions of gene lists (2019 update). *Nucleic Acids Res* 2019;47(W1):W191-W198.

**GSEA**	1) Mootha, V.K., *et al.* PGC-1alpha-responsive genes involved in oxidative phosphorylation are coordinately downregulated in human diabetes. *Nat Genet* 2003;34(3):267-273.<br><br>2) Subramanian, A., *et al.* Gene set enrichment analysis: a knowledge-based approach for interpreting genome-wide expression profiles. *Proc Natl Acad Sci U S A* 2005;102(43):15545-15550.
**GSOAP**	Tokar, T., Pastrello, C. and Jurisica, I. GSOAP: a tool for visualization of gene set over-representation analysis. *Bioinformatics* 2020;36(9):2923-2925.
**PANTHER**	Mi, H., *et al.* PANTHER version 16: a revised family classification, tree-based classification tool, enhancer regions and extensive API. *Nucleic Acids Res* 2021;49(D1):D394-D403.
**REACTOME**	Gillespie, M., *et al.* The reactome pathway knowledgebase 2022. *Nucleic Acids Res* 2022;50(D1):D687-D692.
**REVIGO**	Supek, F., *et al.* REVIGO summarizes and visualizes long lists of gene ontology terms. *PLoS One* 2011;6(7):e21800.
**WebGestalt**	Liao, Y., *et al.* WebGestalt 2019: gene set analysis toolkit with revamped UIs and APIs. *Nucleic Acids Res* 2019;47(W1):W199-W205.

Supplementary Table 1. List of FEA tools featured in Supplementary Figure 1, and their corresponding citation(s) or websites. Tools are listed in alphabetical order.

Supplementary Table 2

Tool	WB [API]/ SA [CL/ GUI]	Flexible[1] – can be used with any database and FEA	Display genes	Display gene-level QD	Highlight gene-sets driving ME, in detail	Highlight patterns in gene-level quantitative data driving ME	Gene-level side-by-side comparison of multiple FEAs[3]	Output enables systematic exploration of FEA results, including gene-level data
**DAVID**	WB [API]	No	Yes	No	Yes	No	No	Yes
**PANTHER**	WB [API]	No	Yes	No	No	No	No	Yes
**REACTOME**	WB [API]	No	Yes	Yes	No	No	No	Yes
**REVIGO**	WB	No	No	No	No	No	No	No
**clusterProfiler**	SA [CL]	Yes	Yes	Yes	No	No	No[3]	No
**EnrichmentMap**	SA [GUI + CL]	Yes	Yes	Yes	No	No	Yes	No
**GeneFEAST**	SA [CL]	Yes	Yes	Yes	Yes	Yes	Yes	Yes
**GOATOOLS**	SA [CL]	No	No	No	No	No	No	No
**GO-Figure!**	SA [CL]	No	No	No	No	No	No	No
**GSEA**	WB + SA [GUI + CL]	No	Yes	Yes	Limited[2]	Limited[2]	No	Yes
**WebGestalt**	WB + SA [CL]	Yes	Yes	No	No	No	No[3]	Yes

Supplementary Table 2. FEA tools that provide a summarisation step, and the additional features that they provide beyond basic summarisation of FEA results. Notes: [1] For a summarisation tool to be fully flexible it must work with terms derived from any database and also be applicable at the end of any FEA pipeline. [2] Using GSEA's 'Leading edge analysis' tool, users are able to view heatmaps of gene expression data that can help identify leading edge genes driving multiple gene-set enrichments. However, although gene-sets are clustered based on their gene-set overlap prior to generating a heatmap, they are not then split into communities before visualisation, rather, one heatmap is generated for all the gene-sets together. This, combined with the fact that genes in the heatmap can only be ordered according to clustering based on their global gene-set membership, with no other sorting options available, means that it is not easy to identify sets of genes or patterns in their quantitative data that drive multiple enrichments; [3] Some FEA summarisation tools, for example clusterProfiler and WebGestalt, offer a side-by-side comparison of multiple FEAs, but on the level of the term rather than on the level of the gene. Here, we are concerned only with those tools that provide the latter. Acronyms: WB, web-based; API, application programming interface; SA, standalone; standalone tools can have two types of interface: CL, command-line; GUI, graphical user interface; QD, quantitative data; ME, multiple enrichments.

Section 2. Finding communities of related terms using their gene-set overlap: algorithm description

Overview

Supplementary Figure 2 gives an overview of the method used by GeneFEAST to group terms into communities of related terms according to their gene-set overlap and, in turn, to group communities into meta communities, also by gene-set overlap.

Supplementary Figure 2

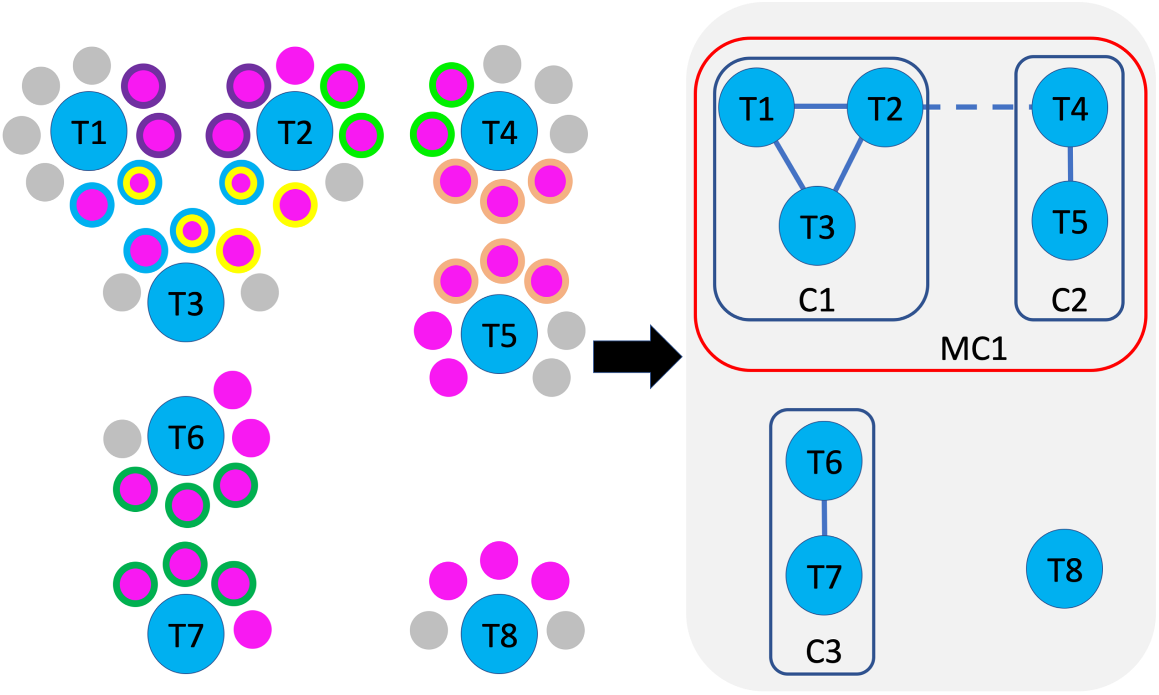

Supplementary Figure 2. Constructing a network of terms using pairwise gene-set overlap, and detecting communities of well-connected terms therein. (Left) Terms are shown as large blue circles, labelled as 'T1', 'T2', etc.; each term is surrounded by its annotated genes, with genes of interest (GoI) coloured pink; genes annotated by more than one term are indicated by matching borders. **(Right)** Terms are connected by an edge, shown as a solid blue line, when their gene-set overlap exceeds a user-defined threshold. Communities of well-connected terms are labelled as 'C1', 'C2', etc., and depicted with blue rectangles. Communities connected by residual gene-set overlaps that did not exceed the edge threshold, depicted as dashed blue lines, are connected into a meta community (MC1), and surrounded by a red rectangle. Such meta communities can also be formed when strong gene-set overlap exists between terms from different databases, but multi-database agglomeration is off (default). 'C3' is a community that is not a member of a meta community. 'T8' is a term that is not a member of a community.

Details

Supplementary Box 1 gives the GeneFEAST algorithm, in pseudocode, for grouping enriched terms identified in one FEA into communities of related terms, according to their gene-set overlap, followed by the pseudocode for finding meta communities of communities. Supplementary Box 2 gives the additional steps required for dealing with terms identified as enriched in multiple FEAs. Supplementary Boxes 3 and 4 give the pseudocode for the attenuation procedure that we apply to greedy modularity maximisation to limit the size of communities and meta communities, respectively.

Supplementary Box 1

Filter out enriched GO terms that are too broad, using maximum descendant threshold and minimum hierarchy level. [1]

Filter out any term annotating too few GoI. [2]

Put all remaining enriched terms in a list called *Enriched_Terms*.

* Create an empty list of communities, called *All_Communities*.

If database agglomeration is off [3]:

Within each database-category of terms:

Extract terms from *Enriched_Terms* that are from the database-category, and put these into a list called *DB_Enriched_Terms*.

Create an empty network of terms called *Term_Network*.

For each pair of enriched terms in *DB_Enriched_Terms*:

Calculate gene-set overlap. [4]

If gene-set overlap exceeds threshold [5]:

Add the pair of terms, with an edge between them, to *Term_Network*.

Find non-singleton communities of terms in *Term_Network* using greedy modularity maximisation [6], attenuated by an adaptive algorithm. (See Supplementary Box 3.) This procedure returns a list of communities called *Communities*. **Concatenate** *Communities to All_Communities*.

Else [7]:

Create an empty network of terms called *Term_Network*

For each pair of terms in *Enriched*_Terms:

Calculate gene-set overlap.

If gene-set overlap exceeds threshold:

Add the pair of terms, with an edge between them, to *Term_Network.*

Find non-singleton communities of terms in *Term_Network* using modularity maximisation, attenuated by an adaptive algorithm. (See Supplementary Box 3.) This procedure returns a list of communities called *Communities*. **Assign** *Communities to All_Communities*.

Identify terms that are not members of a community by calculating:

{term | term ∈ *Enriched_Terms*} − {term | term ∈ $\cup_{i=1}^{N} community_i$},

where N is the total number of communities in *All*_Communities.

Supplementary Box 1, cont.

```
Create an empty network of communities called Community_Network.

For each pair of communities from All_Communities [8]:
     Calculate gene-set overlap. [9]

     If gene-set overlap exceeds threshold [10]:
          Add the pair of communities, with an edge between them, to
          Community_Network.
```

Find non-singleton meta communities of communities in *Community_Network* using modularity maximisation, attenuated by an adaptive algorithm. (See Supplementary Box 4.) This procedure returns a list of meta communities call *Meta_Communities*.

Identify communities that are not members of a meta community by calculating:

{community | community ∈ *All_Communities*} −

{community | community ∈ $\bigcup_{i=1}^{N} meta\_community_i$},

Supplementary Box 1. GeneFEAST algorithm, written in pseudocode, for grouping terms identified as enriched in one FEA into communities of related terms, and for finding meta communities of communities.

Notes:

[1] GeneFEAST uses the Python package GOATOOLS (Klopfenstein, et al., 2018) to assign descendant and hierarchy levels to each GO term. The default settings for the maximum descendant threshold and minimum hierarchy level are 50 and 5, respectively.

[2] The default threshold for the minimum number of GoI is 10.

[3] By default, database agglomeration is off. This means that terms from different databases cannot be placed in the same community; i.e., each community will contain terms from one database only.

[4] Gene-set overlap is calculated using either the either the overlap coefficient (OC) or the Jaccard index (JI), (for two sets, X and Y, $OC = |X \cap Y|/\min(|X|, |Y|)$, and $JC = |X \cap Y|/|X \cup Y|$). By default, GeneFEAST uses the OC.

[5] The default gene-set overlap threshold is 0.5.

[6] GeneFEAST uses the Python package NetworkX (Aric A. Hagberg, 2008) implementation of greedy modularity maximisation (Clauset, et al., 2004).

[7] This branch of the algorithm is followed when the user switches on database agglomeration. When database agglomeration is on, terms from different databases can be placed in the same community.

[8] At this point, whether database agglomeration is on or off, all pairs of communities are inspected for their gene-set overlap, regardless of their contributing databases.

[9] By default, community gene-set overlap is calculated using the Jaccard Index.

[10] The default gene-set overlap threshold is 0.1.

Note that all default settings can be changed by the user, if required.

Supplementary Box 2

```
Filter out enriched GO terms that are too broad using maximum descendant
threshold and minimum hierarchy level.

Filter out any term annotating too few GoI. [1]

Find FEA term-set intersections. [2]

For each FEA term-set intersection:
     Proceed from * in Supplementary Box 1.
```

Supplementary Box 2. GeneFEAST algorithm, written in pseudocode, for dealing with terms identified as enriched in multiple FEAs. When GeneFEAST is used to compare multiple FEAs, the program begins by identifying subsets of terms that are found to be enriched in two or more of the FEAs. We refer to each set of terms found in two or more FEAs as an "FEA term-set intersection". In the simplest case, two FEAs, FEA1 and FEA2, are compared to one another, and the subset of terms identified as enriched in both FEA1 *and* FEA2 are summarised. As a further example, when three FEAs, FEA1, FEA2 and FEA3, are compared to one another, up to four FEA term-set intersections are summarised: (1) those terms identified as enriched in FEA1 and FEA2 only; (2) terms found in FEA2 and FEA3 only; (3) terms found in FEA1 and FEA3 only, and (4), terms identified as enriched in all three FEAs.

Notes:
[1] Here, the set of GoI includes any gene identified as interesting in at least one of the underlying experiments that underwent FEA.
[2] GeneFEAST uses the Python package UpSetPlot (Nothman), to identify FEA term-set intersections.

Supplementary Box 3

Find all communities in *Term_Network* using modularity maximisation.

Make a list, *Communities, of* all communities with
1 < |terms| <= *XL_Threshold*. [1]

Put all communities with |terms| > *XL_Threshold* into a list of communities called *XL_Communities*.

While *XL_Communities* is not empty **and**
((gene-set overlap threshold for adding edge <= 0.9) **or**
(maximum descendant threshold for GO terms >= 10)):

Make a list, *XL*_Terms, of all terms that are members of an XL community (i.e. a community in *XL_Communities*).

Reduce the maximum descendant threshold for GO terms by 5.

Remove any GO terms from *XL_Terms* that exceed this threshold.

Build a network of terms out of the remaining terms.
(See Supplementary Box 1, blue sub-box.)

Find communities using modularity maximisation.

Add any communities with
1 < |terms| <= *XL_Threshold* to *Communities*.

XL_Communities is updated to contain communities for which |terms| > *XL_threshold*.

Again, make a list, *XL*_Terms, of all terms that are members of an XL community.

Increase the gene-set overlap threshold for adding an edge by 0.1

Using this new threshold, build a network of terms out of the remaining terms. (See Supplementary Box 1, blue sub-box.)

Find communities using modularity maximisation.

Add any communities with
1 < |terms| <= *XL_Threshold* to *Communities*.

XL_Communities is emptied and then updated to contain communities for which |terms| > *XL_threshold*.

Add *XL_Communities* to *Communities* [2].
Return *Communities*

Supplementary Box 3. GeneFEAST algorithm, written in pseudocode, for the attenuation procedure applied to greedy modularity maximisation to limit the size of communities.

Notes:

[1] *XL_Threshold* is the preferred maximum size for a community, with communities larger than this threshold considered extra-large (hence 'XL'). The default value is 15, but the user can change this, if required. Note that the algorithm will attempt to break up extra-large communities, but that this may not always work, which is why the maximum size is 'preferred' rather than 'guaranteed'.

[2] If the end of the procedure is reached and there remain extra-large communities, these are added to the list of communities arising from those extra-large communities that have been successfully broken up.

Supplementary Box 4

```
Find all communities in Community_Network using modularity maximisation.
These are meta communities of communities.

Make a list, Meta_Communities, of all meta communities with
1 < |communities| <= XL_Threshold. [1]

Put all meta communities with |communities| > XL_Threshold into a list
of meta communities called XL_Meta_Communities.

While XL_Meta_Communities is not empty and
  (gene-set overlap threshold for adding edge <= 0.9):

     Make a list, XL_Communities, of all communities that are members
     of an XL meta community.

     Increase the gene-set overlap threshold for adding an edge by 0.1.

     Using this new threshold, build a network of communities out of
     the communities. (See Supplementary Box 1, red sub-box.)

     Find meta communities using modularity maximisation.

     Add any meta communities with
     1 < |communities| <= XL_Threshold to Meta_Communities.

     XL_Meta_Communities is emptied and updated to contain meta
     communities for which |communities| > XL_Threshold.

Add XL_Meta_Communities to Communities [2].
Return Meta_Communities
```

Supplementary Box 4. GeneFEAST algorithm, written in pseudocode, for the attenuation procedure applied to greedy modularity maximisation to limit the size of meta communities.

Notes:

[1] `XL_Threshold` is the preferred maximum size for a meta community, with meta communities larger than this threshold considered extra-large (hence 'XL'). The default value is 15, but the user can change this, if required. Note that the algorithm will attempt to break up extra-large meta communities, but that this may not always work, which is why the maximum size is 'preferred' rather than 'guaranteed'.

[2] If the end of the procedure is reached and there remain extra-large meta communities, these are added to the list of meta communities arising from those extra-large meta communities that have been successfully broken up.

Supplementary Figure 3. Silhouette plot of communities

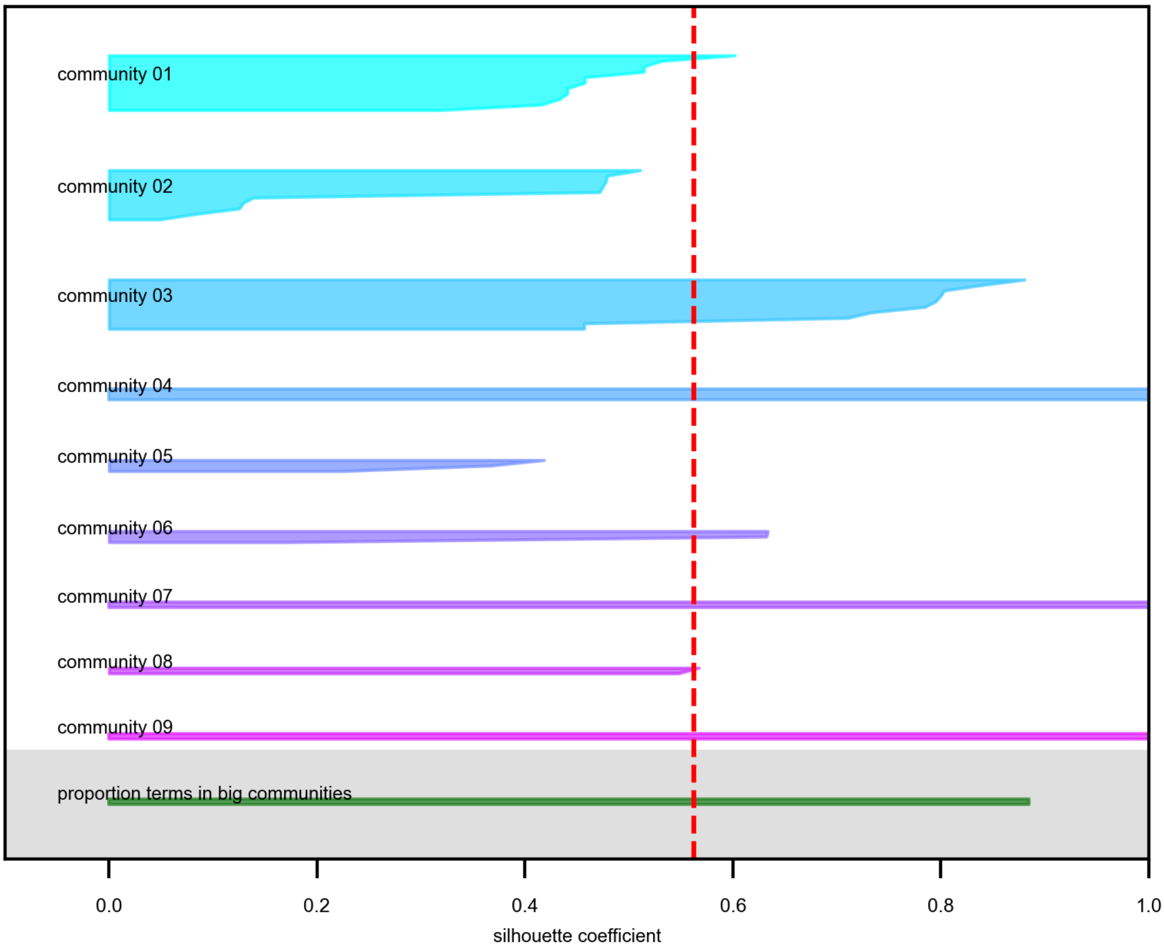

Supplementary Figure 3. Silhouette plot of communities. An example silhouette plot generated by GeneFEAST. This silhouette plot was generated when GeneFEAST was used to summarise a FEA conducted on publicly available data (Pinto, et al., 2023) and nine communities of terms were identified. The red dashed vertical line indicates the mean silhouette score obtained over all terms. Added to the bottom of the silhouette plot is a green bar on a grey background indicating the proportion of terms identified as being part of any community containing two or more terms. In this example, ~90% of terms are in a community, meaning that ~10% of terms are singleton and have not been included in a community of two or more terms.

Supplementary Figure 4. Graphical grid search of community detection parameters

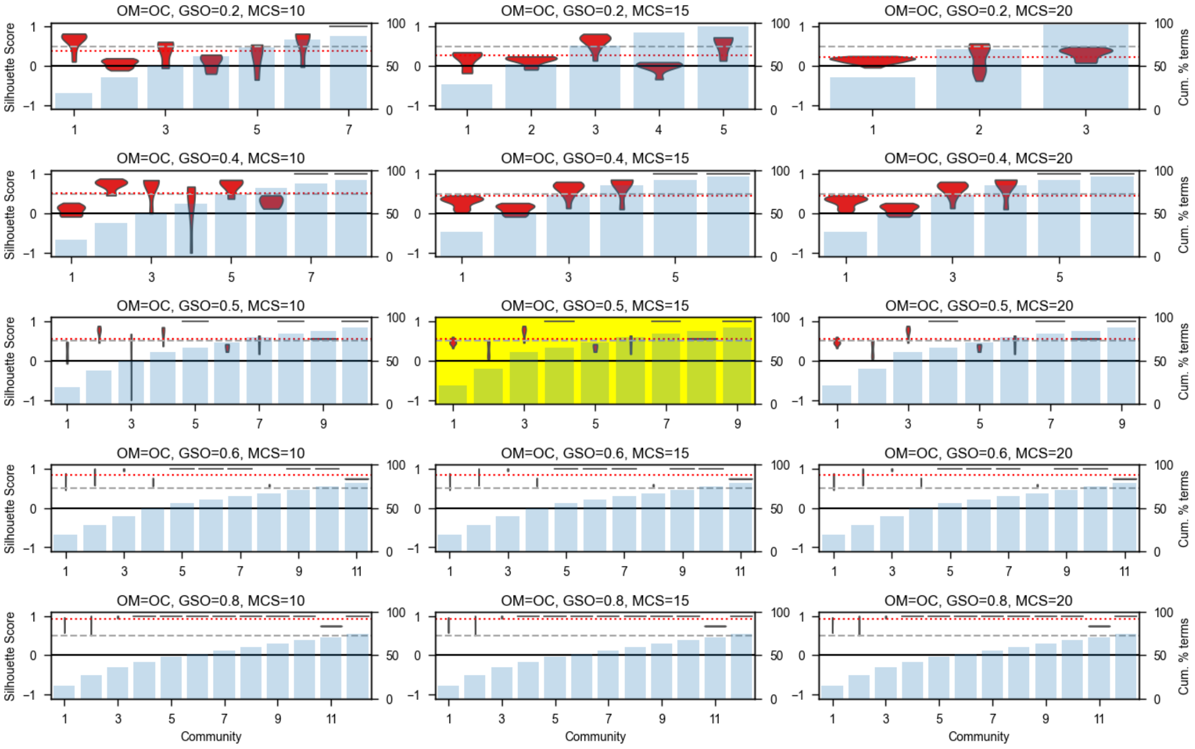

Supplementary Figure 4. Graphical grid search of community detection parameters. An example graphical grid search of community detection parameters generated by GeneFEAST. This graphical grid search was generated when GeneFEAST was used to summarise an FEA conducted on publicly available data (Pinto, et al., 2023) and nine communities of terms were identified. The graphical grid search is comprised of subplots such that each subplot characterises the communities obtained for a given tuple of overlap measure, gene-set overlap threshold and maximum community size; the title of each subplot indicates the tuple of values used (OM = overlap measure, GSO = gene-set overlap, and MCS = maximum community size). Within each subplot, the x-axis indicates how many communities of terms were obtained with this tuple of values, with one bar/ violin plot pair being plotted per community. The violin plots are plotted against the left-hand y-axis and show the distribution of silhouette scores obtained for terms in each community; the red dotted line then shows the mean silhouette score obtained over all terms, and the black dashed line indicates a silhouette score of 0.5 (for reference). Underneath these violin plots are bars, plotted against the right-hand y-axis, that show the cumulative percentage of terms included in communities. GeneFEAST produces separate graphical grid searches for each of the two overlap measures available (Overlap Coefficient (OC) and Jaccard Index (JI)). The graphical grid searches always include a subplot for the overlap measure, gene-set overlap and maximum community size thresholds set by the user, and the corresponding subplot is highlighted with a yellow background.

References

Aric A. Hagberg, D.A.S.a.P.J.S. Exploring network structure, dynamics, and function using NetworkX. *Proceedings of the 7th Python in Science Conference (SciPy2008)* 2008.

Clauset, A., Newman, M.E. and Moore, C. Finding community structure in very large networks. *Phys Rev E Stat Nonlin Soft Matter Phys* 2004;70(6 Pt 2):066111.

Klopfenstein, D.V.*, et al.* GOATOOLS: A Python library for Gene Ontology analyses. *Sci Rep* 2018;8(1):10872.

Nothman, J. UpSetPlot. https://upsetplot.readthedocs.io/en/stable/index.html.

Pinto, S.M., *et al*. Multi-OMICs landscape of SARS-CoV-2-induced host responses in human lung epithelial cells. iScience. 2023 Jan 20;26(1):105895.
